# Integrated Fault Estimation and Fault-Tolerant Control for Drive-by-Wire Steering Systems in Four-Wheel Independent Drive Vehicles

**DOI:** 10.3390/s26134185

**Published:** 2026-07-02

**Authors:** Feng Wang, Wangcheng Chu, Xiaoyuan Zhu

**Affiliations:** 1 Automotive Engineering Research Institute, Jiangsu University, Xuefu Road, Zhenjiang 212013, China; bewater@ujs.edu.cn (F.W.); 2232338003@stmail.ujs.edu.cn (W.C.); 2 School of Mechanical Engineering, Southeast University, Southeast University Road, Nanjing 211189, China

**Keywords:** integrated fault estimation and fault-tolerant control, steer-by-wire, actuator faults, unknown input observer

## Abstract

This study proposes an integrated fault estimation and fault-tolerant control strategy for actuators of four-wheel independently driven vehicles. Firstly, a modeling approach is developed by combining the vehicle dynamics model with the steering actuator model to establish a system model in the presence of actuator faults. Subsequently, to simultaneously estimate the system states and actuator faults, an unknown input observer (UIO) is designed, treating actuator faults as unknown state variables and augmenting the system state variables to achieve simultaneous estimation of states and faults, providing a foundation for subsequent fault-tolerant control research. The gain matrices of the fault observer and the fault-tolerant controller are both obtained through single-step linear matrix inequality (LMI) computations, realizing the design of an integrated fault-tolerant control system. Finally, the effectiveness and superiority of the proposed method are verified through hardware-in-the-loop experimental tests.

## 1. Introduction

Driven by the fast-paced development of intelligent automotive technology, the steer-by-wire (SBW) system, integral to four-wheel independently driven drives, has become a critical area of investigation [1,2,3]. Unlike traditional designs, the mechanical linkage between the steering column and the steering gear is eliminated by the SBW system, instead achieving active steering functionality by driving the steering motor. It has advantages such as short response time and high control accuracy [4,5]. Nevertheless, the incorporation of electronic elements—including sensors, controllers, and steering motors—raises the probability of system faults, presenting significant hurdles to vehicle reliability [6]. Among these, actuators are particularly prone to failure, given their complex operational mechanisms. Should an actuator malfunction occur, it would directly compromise the vehicle’s maneuverability and handling stability, potentially giving rise to critical safety risks. Therefore, under these circumstances, effective fault-tolerant control is essential [7,8]. To address actuator faults during vehicle operation that compromise operational reliability, this paper investigates fault-tolerant control (FTC) strategies for actuator faults in SBW systems [9].

Regarding fault-tolerant control for vehicles, many investigations have been undertaken by scholars. Zhang et al. addressed the challenge of actuator faults in steer-by-wire systems by devising a coordinated control strategy that combines differential steering with direct yaw moment to safeguard trajectory tracking and yaw stability [10]. Liu et al., focusing on nonlinear systems within a certain class subject to actuator faults, proposed an integrated robust real-time FTC design method employing linear matrix inequalities [11]. Huang et al. proposed an FTC method for SBW systems with actuator faults and finite uncertainties, designing a fault observer to evaluate fault signal and the fault SBW system under fault conditions [12]. Huang et al. presented a fault-tolerant control method for SBW systems using model predictive control (MPC). This approach utilizes a triangular operator to construct a fault observer and then employs MPC to compensate for actuator faults, thereby maintaining the vehicle’s tracking performance [13]. Sun et al., focusing on actuator attack scenarios, examined a data-driven event-triggered secure adaptive control (DET-ASC) approach for autonomous vehicle (AV) lateral regulation [14]. Boudaoud et al., focusing on steering actuator faults in SbW systems for driver assistance, developed a fault-tolerant shared control (FTSC) scheme using an LPV observer-based adaptive state feedback controller with a Takagi–Sugeno (T-S) approach, formulated as an LMI optimization problem to ensure stability [15]. Sentouh et al., focusing on unknown bounded actuator faults in integrated lateral and longitudinal vehicle control systems, developed an interconnected LPV observer-based adaptive fault estimation and control reconfiguration framework [16]. Meléndez-Useros et al., focusing on steering actuator faults in autonomous vehicles, developed a fault-tolerant path-tracking static output-feedback controller using a Linear Parameter Varying (LPV) approach [17].

Although existing research has proposed various fault-tolerant control methods for actuator faults in steer-by-wire systems, the fault-tolerant control scheme commonly used in the existing steer-by-wire system is mainly designed around steering angle tracking. Usually, the steering angle command is used as the only input, ignoring the internal dynamics of the motor current. However, our proposed method uses steering motor current as a direct driving variable to achieve faster and more accurate fault compensation. In addition, most previous SBW FTC studies are still limited to front wheel steering adjustment, and direct yaw moment control is rarely integrated to actively maintain vehicle lateral stability. In order to solve this problem, this study takes DYC as an integral part of the control architecture. Through fault-tolerant torque redistribution, the yaw moment is dynamically redistributed among the four in-wheel motors to maintain vehicle stability under the guidance of real-time fault estimation provided by the unknown input observer.

However, due to the interaction between the FTC and fault estimation (FE) system functions, a complex coupling of uncertainties inevitably arises between them. This mutual coupling implies that FE and FTC models developed independently may be incompatible when implemented simultaneously. Ding et al. emphasized that the FE and FTC/FTTC modules require joint design to attain satisfactory control performance [18]. Lan and Patton examined the bidirectional robust interactions between FE and FTC/FTTC modules and introduced an effective integrated FE/FTC strategy tailored for uncertain linear systems [19]. Zhao et al. introduced a combined FE and fault-tolerant tracking control methodology for Lyapunov non-linear multi-agent systems with actuator faults, external disturbances, and parametric uncertainties occurring concurrently [20]. Cheng et al. presented an integrated fault estimation and fault-tolerant control (integrated FE and FTC) scheme for time-delay uncertain Lyapunov systems operating within a finite state space [21]. Liu et al. presented an active reconfigurable controller aimed at mitigating actuator fault consequences in flexible aircraft. Building on this, they proposed a novel integrated FE and FTC methodology to resolve actuator fault issues [22]. Although references [18,19,20,21,22] have made achievements in integrated fault-tolerant control of aircraft or theoretical systems, they have rarely been applied to the vehicle field, and the integrated design has not yet been implemented in vehicle tolerance control.

The traditional fault-tolerant control architecture regards observer design and controller synthesis as independent modules. However, this separation introduces a two-way defect: the estimation error will reduce the control effect, and the control behavior will affect the estimation accuracy. In order to overcome these limitations and improve the overall FTC performance, this paper treats fault estimation and fault-tolerant regulation as a coupled design problem. In order to solve the steering actuator failure that threatens the lateral stability of the vehicle, we designed an integrated framework to jointly optimize the observer gain and controller gain through a one-step LMI method. Different from the decoupling method, the proposed scheme improves the estimation accuracy, response speed and robustness. The experimental verification shows that the integrated scheme accelerates the fault detection speed and improves the fault tolerance accuracy, thus effectively suppressing the vehicle instability caused by the steering system fault. The principal contributions of this research can be outlined as shown below:

(1) For vehicle systems with actuator faults, modeling that considers the execution characteristics of the steering system can more accurately reflect the dynamic characteristics of actuator faults.
(2) For vehicle systems with actuator faults, an integrated FE and FTC design method is formulated, which relies on single-step linear matrix inequalities (LMIs).
(3) Through offline simulation and controller-in-the-loop simulation, the proposed control strategy was validated for effectiveness under actuator fault conditions. Results demonstrate that this strategy reliably ensures vehicle driving stability.

This study is structured in the following manner: In Part II the vehicle dynamics model and the SBW system model are described. Part III introduces the integrated FE and FTC system, comprising four sections: integrated design, along with the description of the system, the design of FE and the design of FTC. Part IV presents the experimental results. Finally, a comprehensive analysis and discussion of the integrated FE and FTC scheme for SBW system faults is provided, along with conclusions and directions for subsequent research.

## 2. System Modeling

### 2.1. Vehicle Dynamics Model Construction

To facilitate reference model design while preserving the dominant characteristics of vehicle lateral dynamics, the classical two-degree-of-freedom monorail vehicle model is used as the reference model to describe the lateral motion and yaw motion of the vehicle. The model simplifies the vehicle as a rigid body with front and rear axles, ignores the suspension, pitch and roll motions, and only considers the two degrees of freedom of sideslip angle and yaw rate, which can effectively describe the lateral dynamic response of the vehicle under steering input.

**Figure 1:**
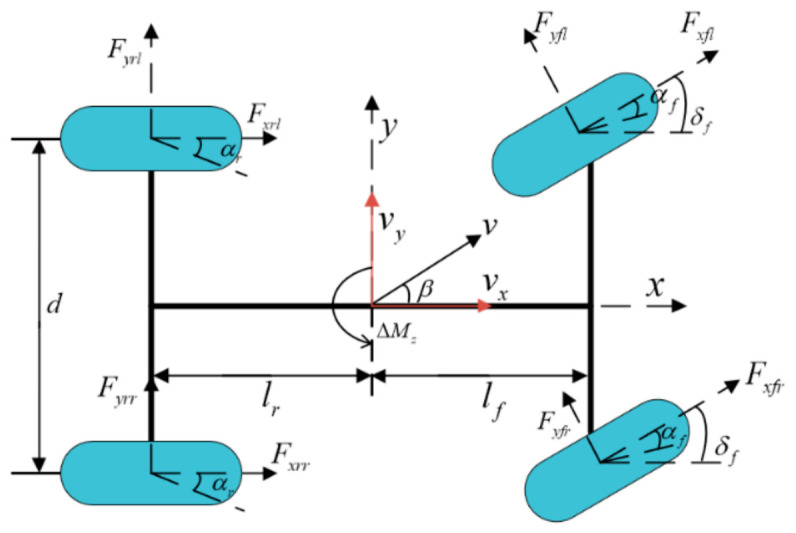
Vehicle dynamics model.

Figure 1 presents the adopted two-degree-of-freedom vehicle dynamic model, whose governing equations take the following form:

(1) $$\left\{\begin{array}{l} \dot{\beta }=-\frac{2(C_{f}+C_{r})}{mv_{x}}\beta -(1+\frac{2(l_{f}C_{f}-l_{r}C_{r})}{mv_{x}^{2}})\dot{\psi }+\frac{2C_{f}}{mv_{x}}\delta \\ \ddot{\psi }=-\frac{2(l_{f}C_{f}-l_{r}C_{r})}{I_{z}}\beta -\frac{2(l_{f}^{2}C_{f}+l_{r}^{2}C_{r})}{I_{z}v_{x}}\dot{\psi }+\frac{2l_{f}C_{f}}{I_{z}}\delta +\frac{1}{I_{z}}\Delta M_{z} \end{array}\right.$$

Among them, the state quantity is ${\left[\begin{array}{cc} \beta & \dot{\psi } \end{array}\right]}^{T}$; the control input is ${\left[\begin{array}{cc} \delta & \Delta M_{z} \end{array}\right]}^{T}$.

Among them β and φ denote the center of mass sideslip angle and yaw-rate, respectively; the vehicle mass is denoted by m; the lateral stiffness of the front and rear tires are represented by $C_{f}$ and $C_{r}$; $\delta_{f}$ corresponds to the angle at which the driver steers the front wheels; and $\Delta \delta_{f}$ refers to the correction steering angle. The actual front wheel steering angle, expressed as δ, is obtained by summing $\delta_{f}$ and $\Delta \delta_{f}$. The moment of inertia associated with the vehicle relative to the z-direction axis is denoted by $I_{z}$, where the lengths between the center of gravity and the front/rear axles of gravity are represented by $l_{f}$ and $l_{r}$, respectively. $\Delta M_{z}$ indicates the direct yaw moment, which takes the form:

(2) $$\Delta M_{z}=d(F_{xrl}+F_{xrr}-F_{xfl}-F_{xfr})/2$$

$v_{x}$ and $v_{y}$ denote the longitudinal and lateral velocities of the vehicle; the tire sideslip angles for the front and rear wheels are represented by $\alpha_{f}$ and $\alpha_{r}$; the wheel track is denoted by d; ij=fl,fr,rl,rr respectively represent the vehicle’s four wheels; and $F_{xij},F_{yij}$ represent the longitudinal and lateral forces of each wheel, decomposed and combined in the vehicle coordinate system.

### 2.2. Steer-by-Wire Steering Actuator Model

**Figure 2:**
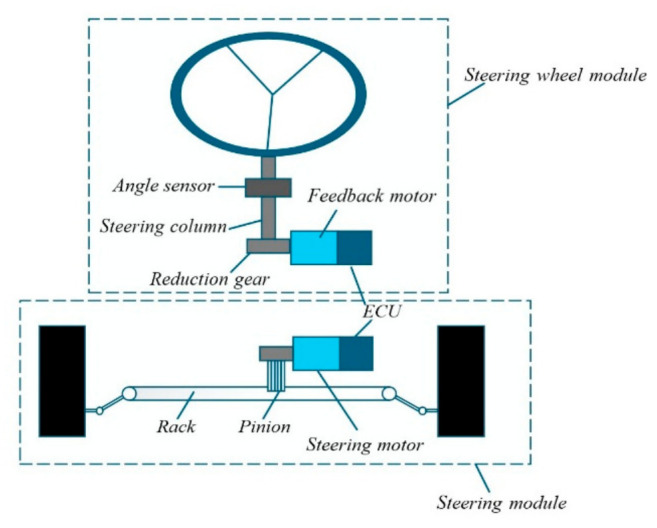
The schematic of SBW system.

As shown in Figure 2, the SBW system consists of a steering motor, a rack-and-pinion mechanism, a steering trapezoidal mechanism, and steerable front wheels [23,24,25]. The steering motor serves as the actuator of the SBW system, providing torque to drive the front wheels for steering.

The dynamic equations for the steering motor are presented below [26]:

(3) $$J_{eq}\ddot{\delta }+B_{eq}\dot{\delta }+C_{eq}\delta =K_{pw}\cdot T_{in}+2\tau_{a}$$

where the equivalent moments of inertia, damping, and stiffness of the steering actuator are denoted by $J_{eq}$, $B_{eq}$ and $C_{eq}$; the transmission ratio between the steering actuator motor and rack and pinion is denoted by $K_{pw}$; the input torque acting on the steering gear is denoted by $T_{in}$; and the backward correction torque of the left and right front wheels about their respective kingpins is denoted by $\tau_{a}$, represented as

(4) $$\tau_{a}=-(t_{p}+t_{m})\cdot F_{yf}=-(t_{p}+t_{m})\cdot C_{f}(\delta -\beta -\frac{l_{f}\dot{\psi }}{v_{x}})$$

Among them, $t_{p}$ and $t_{m}$ are known as the pneumatic, as well as the mechanical, trail. Define $T_{in}$ as the steering actuator output torque, which can be written as:

(5) $$T_{in}=k_{in}\cdot i$$

With $k_{in}$ defined as the output torque parameter and the motor current of the steering actuator as i.

Presented below is the equation describing the dynamic characteristics pertaining to the steering actuator:

(6) $$\begin{array}{ll} \ddot{\delta } & =-\frac{B_{eq}}{J_{eq}}\dot{\delta }-\frac{2\left(t_{p}+t_{m}\right)C_{f}+C_{eq}}{J_{eq}}\delta \\ & +\frac{2\left(t_{p}+t_{m}\right)C_{f}}{J_{eq}}\beta +\frac{2\left(t_{p}+t_{m}\right)C_{f}l_{f}}{J_{eq}v_{x}}\psi +\frac{k_{pw}k_{in}}{J_{eq}}i \end{array}$$

Based on the vehicle and SBW actuator models, a joint dynamic model is constructed for state estimation, as shown in the following equation:

(7) $$\left\{\begin{array}{l} \begin{array}{ll} \ddot{\delta } & =-\frac{B_{eq}}{J_{eq}}\dot{\delta }-\frac{2\left(t_{p}+t_{m}\right)C_{f}+C_{eq}}{J_{eq}}\delta +\frac{2\left(t_{p}+t_{m}\right)C_{f}}{J_{eq}}\beta \\ & +\frac{2\left(t_{p}+t_{m}\right)C_{f}l_{f}}{J_{eq}v_{x}}\dot{\psi }+\frac{k_{pw}k_{in}}{J_{eq}}i \end{array}\\ \dot{\delta }=\dot{\delta }\\ \dot{\beta }=\frac{2C_{f}}{mv_{x}}\delta -\frac{2(C_{f}+C_{r})}{mv_{x}}\beta -(1+\frac{2(l_{f}C_{f}-l_{r}C_{r})}{mv_{x}^{2}})\dot{\psi }\\ \ddot{\psi }=\frac{2l_{f}C_{f}}{I_{z}}\delta -\frac{2\left(l_{f}C_{f}-l_{r}C_{r}\right)}{I_{z}}\beta -\frac{2\left(l_{f}^{2}C_{f}+l_{r}^{2}C_{r}\right)}{I_{z}v_{x}}\dot{\psi }+\frac{1}{I_{z}}\Delta M_{z} \end{array}\right.$$

Let $\dot{\delta }$, δ, β, $\dot{\psi }$ represent the state quantity of the steer-by-wire system. Among the variables, $\dot{\delta }$ is the derivative of the front wheel rotation angle, δ is the front wheel angle, β and $\dot{\psi }$ represent the sideslip angle and yaw rate of the vehicle respectively, $u={\left[i\;\Delta M_{z}\right]}^{T}$ represents the control input of steer-by-wire system, i is the steering motor current, and $\Delta M_{z}$ is the vehicle yaw moment.

### 2.3. Tire Model

Tires serve as the exclusive physical link between the vehicle and the road surface, channeling nearly all external loads encountered during vehicle motion to the chassis. The mechanical responses of tires exhibit pronounced variations across diverse operational scenarios. This is especially true under challenging conditions—including high-adhesion road surfaces, high-speed turning maneuvers, and low-speed travel on slippery pavements—where the tire forces frequently extend into the nonlinear regime. The fidelity of tire modeling substantially influences the dependability of simulation outcomes, which in turn shapes the precision of subsequent vehicle dynamic assessments. Consequently, the development of accurate tire models carries considerable weight in both theoretical inquiries into vehicle dynamics and practical engineering implementations.

To enhance the robustness of the proposed controller under challenging driving scenarios, particularly high-speed operation on low-adhesion surfaces, this study employs the Magic Formula tire model for lateral force computation. This semi-empirical model reconstructs tire test data through a composite of trigonometric functions, yielding an accurate mathematical representation of tire force characteristics. The Magic Formula is widely recognized for its structurally unified formulation and superior fitting precision. Even under severe conditions involving substantial longitudinal slip or large sideslip angles, the model maintains satisfactory accuracy and broad applicability. The resulting lateral force under pure cornering conditions is expressed as follows:

(8) $$F_{y}=D_{y}\;\sin\{C_{y}\;\arctan[B_{y}\alpha -E_{y}(B_{y}\alpha -\arctan(B_{y}\alpha ))]\}$$

Among the variables, *C_y_ = b*_0_, *D_y_ = b*_1_*F_z_*^2^ + *b*_2_*F_z_*, *B_y_ = b*_3_*sin*(*b*_4_*arctan*(*b*_5_*F_z_*))/(*C_y_D_y_*), *E_y_ = b*_6_*F_z_*^2^ + *b*_7_*F_z_ + b*_8_, F_y_ is the wheel lateral force, α is the tire cornering angle, and b0~b8 are the fitting coefficients of the tire model. The specific values are shown in Table 1.

The lateral acceleration generated by the turning of the vehicle will cause the load redistribution between the left and right wheels. Based on this dynamic effect, the actual vertical load on each tire can be expressed as

(9) $$\left\{\begin{array}{c} F_{zfl}=\frac{mgl_{r}}{2L}-\frac{ma_{y}h_{g}l_{r}}{LB_{f}}\\ F_{zfr}=\frac{mgl_{r}}{2L}+\frac{ma_{y}h_{g}l_{r}}{LB_{f}}\\ F_{zrl}=\frac{mgl_{f}}{2L}-\frac{ma_{y}h_{g}l_{f}}{LB_{r}}\\ F_{zrr}=\frac{mgl_{f}}{2L}+\frac{ma_{y}h_{g}l_{f}}{LB_{r}} \end{array}\right.$$

In the formula, F_zfl_, F_zfr_, F_zrl_ and F_zrr_ represent the vertical loads of the left front wheel, the right front wheel, the left rear wheel and the right rear wheel respectively. a_y_ is the lateral acceleration of the vehicle; b_f_ and B_r_ represent the front and rear wheelbase respectively; and h_g_ is the height of the center of mass.

Considering the effects of longitudinal acceleration and yaw moment on vertical wheel load transfer, torque distribution has therefore been optimized. The lateral force for each wheel $F_{xi}$ is given by the following formula:

(10) $$\left\{\begin{array}{c} F_{xfl}=\frac{mgl_{r}}{2gL}(\sum F-\frac{2\Delta M_{z}}{d})\\ F_{xfr}=\frac{mgl_{r}}{2gL}(\sum F+\frac{2\Delta M_{z}}{d})\\ F_{xrl}=\frac{mgl_{f}}{2gL}(\sum F-\frac{2\Delta M_{z}}{d})\\ F_{xrr}=\frac{mgl_{f}}{2gL}(\sum F+\frac{2\Delta M_{z}}{d}) \end{array}\right.$$

where ax is the longitudinal acceleration and d is the distance between the two wheels on the coaxial. ∑F corresponds to the total drive torque generated by the vehicle.

The value of each wheel torque distribution can be calculated [27] using the following equation:

(11) $$T_{i}=F_{xi}R$$

Let R be the effective radius of the wheel, where i=fl,fr,rl,rr.

The following is the state-space form of the SBW system:

(12) $$\left\{\begin{array}{l} \dot{x}=Ax+Bu\\ y=Cx \end{array}\right.$$

$$A=\left[\begin{array}{cccc} -\frac{B_{eq}}{J_{eq}} & -\frac{2(t_{p}+t_{m})C_{f}+C_{eq}}{J_{eq}} & \frac{2(t_{p}+t_{m})C_{f}}{J_{eq}} & \frac{2(t_{p}+t_{m})C_{f}l_{f}}{J_{eq}v_{x}}\\ 0 & 1 & 0 & 0\\ 0 & \frac{2C_{f}}{mv_{x}} & 1-\frac{2(C_{f}+C_{r})}{mv_{x}} & -(1+\frac{2(l_{f}C_{f}-l_{r}C_{r})}{mv_{x}^{2}})\\ 0 & \frac{2l_{f}C_{f}}{I_{Z}} & -\frac{2(l_{f}C_{f}-l_{r}C_{r})}{I_{Z}} & 1-\frac{2(l_{f}^{2}C_{f+}l_{r}^{2}C_{r})}{I_{Z}v_{x}} \end{array}\right];\;B=\left[\begin{array}{ll} \frac{k_{pw}k_{in}}{J_{eq}} & 0\\ 0 & 0\\ 0 & 0\\ 0 & \frac{1}{I_{z}} \end{array}\right];\;x=\left[\begin{array}{c} \dot{\delta }\\ \delta \\ \beta \\ \dot{\psi } \end{array}\right]$$

### 2.4. Fault Model for SBW System Actuator

In this document, actuator’s fault refers to the actuator’s inability to perform its intended action. When an actuator malfunctions, the SBW system fails to achieve the desired steering assist effect. Actuator faults can be categorized into three main types: gain variation faults (multiplicative faults), constant deviation faults (additive faults), and stuck faults (actuator with constant output). Stuck faults include two types: complete failure and runaway conditions.

Fault outputs for different categories of actuators are expressed uniformly as [28]:

(13) $$u_{q}=\beta u+u_{a}$$

where β indicates the actuator gain variation coefficient, $u_{a}$ denotes the value indicating faults such as constant deviation or sticking, and u denotes the output of the normally functioning actuator.

In this article, in order to verify the effectiveness of the proposed integrated FE/FTC framework, the sinusoidal time-varying fault is selected as the representative condition in the experimental part. The sinusoidal fault has the characteristics of both amplitude change and bias change, which is a comprehensive test of the dynamic tracking ability of the observer and the dynamic compensation ability of the controller. If the method can achieve good results under sinusoidal faults, it has a conservative guarantee for constant deviation and gain variation faults.

The actuator fault model of the SBW system is represented as shown below:

(14) $$\left\{\begin{array}{l} \dot{x}=Ax+B\bar{u}+Bf_{a}\\ y=Cx \end{array}\right.$$

Among the variables, $A\in R^{4\times 4}$, $B\in R^{4\times 2}$, $C\in R^{4\times 4}$ are known constant matrices, $\bar{u}=\Delta u+u_{0}$, $\bar{u}$ is the computational load of the control unit, Δu is the controller output, $u_{0}$ is the feedforward compensation term, and $f_{a}\in R^{2}$ is the actuator fault vector.

## 3. Integrated Fault Estimation and Fault-Tolerant Control

**Figure 3:**
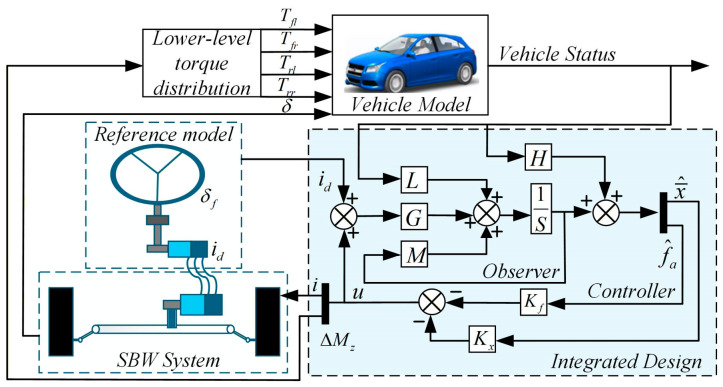
Strategy design for integrated FE and FTC.

Figure 3 provides a schematic diagram of the integrated FE and FTC strategies designed for vehicle actuator faults.

We treat the actuator’s fault as assistive states, thereby augmenting system (14) into:

(15) $$\left\{\begin{array}{l} \dot{\bar{x}}=\bar{A}\bar{x}+\bar{B}\bar{u}\\ y=\bar{C}\bar{x} \end{array}\right.$$

Among the variables, $\bar{x}=\left[\begin{array}{c} x\\ f_{a} \end{array}\right]$, $\bar{A}=\left[\begin{array}{cc} A & B\\ 0 & 0 \end{array}\right]$, $\bar{B}=\left[\begin{array}{c} B\\ 0 \end{array}\right]$, and $\bar{C}=\left[\begin{array}{cc} C & 0 \end{array}\right]$.

**Remark** **1.** *Given the observability of the matrix pair (A, C), systems (14) and (15) are equivalent in terms of observability.*

$$rank\left[\begin{array}{c} sI_{n}-A\\ C \end{array}\right]=n,\;\forall s\in C,\;Re(s)\geq 0$$

*Thus, we obtain the following:*

$$rank\left[\begin{array}{c} sI_{n}-\bar{A}\\ \bar{C} \end{array}\right]=rank\left[\begin{array}{cc} sI_{n}-A & B\\ 0 & sI_{q}\\ C & 0 \end{array}\right]=n+q,\;\forall s\in C,\;Re(s)\geq 0$$

### 3.1. Fault Estimator Design

The extended state $\bar{x}$ is obtained by observing the unknown input through an observer, as given by Equation (16).

(16) $$\left\{\begin{array}{l} \dot{z}=Mz+G\bar{u}+Ly\\ \hat{\bar{x}}=z+Hy \end{array}\right.$$

Here, $z\in R^{6}$ is the observed state of the system and $\hat{\bar{x}}\in R^{6}$ is the estimated value of $\bar{x}$, where M, G, L, and H are matrices to be solved.

Defining the estimation error as $e=\bar{x}-\hat{\bar{x}}$, the derivative of the estimation error is as shown below:

(17) $$\begin{array}{ll} \dot{e} & =\dot{\bar{x}}-\dot{\hat{\bar{x}}}\\ & =(\Xi \bar{A}-L_{1}\bar{C})e+(\Xi B-G)\bar{u}\\ & \;+[(\Xi \bar{A}-L_{1}\bar{C})H-L_{2}]y+(\Xi \bar{A}-L_{1}\bar{C}-M)z \end{array}$$

where $\Xi =I_{6}-H\bar{C}$ and $L=L_{1}+L_{2}$. The matrices M, N, G and $L_{2}$ are defined as

(18) $$M=\Xi \bar{A}-L_{1}\bar{C},\;N=\Xi ,\;G=\Xi \bar{B},\;L_{2}=(\Xi \bar{A}-L_{1}\bar{C})H$$

Based on the definition specified in Equation (18), the dynamic error (17) can be expressed as

(19) $$\dot{e}=(\Xi \bar{A}-L_{1}\bar{C})e$$

**Remark** **2.** *There exist matrices H and L_1_ such that* $\Xi \bar{A}-L_{1}\bar{C}$ *is the Hurwitz matrix, where* $\Xi =I_{n+q}-H\bar{C}\;$ *. This condition suffices to ensure the asymptotic stability of the error dynamic system.
*

### 3.2. Fault-Tolerant Controller Design

Define the system’s fault-tolerant controller as

(20) $$\Delta u=-K\hat{\bar{x}}$$

where $K=\left[\begin{array}{cc} K_{x} & K_{f} \end{array}\right]$, $K_{x}\in R^{2\times 4}$, and $K_{f}\in R^{2\times 2}$.

Substituting Equation (20) into Equation (12) yields:

(21) $$\dot{x}=(A-BK_{x})x+BKe+Bu_{0}$$

Let $w=u_{0}$, then

(22) $$\dot{x}=(A-BK_{x})x+BKe+Bw$$

### 3.3. FE/FTC Integrated Design

The augmented closed-loop system can be represented by Equations (19) and (22):

(23) $$\left\{\begin{array}{l} \dot{x}=(A-BK_{x})x+BKe+Bw\\ \dot{e}=(\Xi \bar{A}-L_{1}\bar{C})e \end{array}\right.$$

Define the objective control of the fault-tolerant controller as

(24) $$o=C_{x}x+C_{e}e=\left[\begin{array}{cc} C_{x} & C_{e} \end{array}\right]\left[\begin{array}{c} x\\ e \end{array}\right]$$

where $C_{x}\in R^{4\times 4},C_{e}\in R^{4\times 6}$.

With the inclusion of this control objective, the system state and the estimation error can be driven to converge concurrently, which realizes a truly unified framework for fault estimation and fault-tolerant control.

The *H*_∞_ performance of the fault-tolerant controller for SBW system is defined as:

(25) $${∥T_{ow}∥}_{\infty }=\sup_{{∥w∥}_{2\neq 0}}\frac{{∥o∥}_{2}}{{∥w∥}_{2}}$$

where ${∥o∥}_{2}$ is the 2-norm of the control target signal ∥o∥ and ${∥w∥}_{2}$ is the 2-norm of w. The bounded real lemma shows that the SBW system is stable and ${∥T_{ow}∥}_{\infty }$ is the smallest.

Equation (23) reveals that estimation errors directly affect the control system. This observation points to a bidirectional robust coupling between the FE and FTC modules, highlighting the necessity of an integrated FE and FTC design methodology to guarantee optimal stability at the system level.

Define the Lyapunov function as

(26) $$V=x^{T}Px+e^{T}P_{1}e$$

where $P=P^{T}>0,P_{1}=P_{1}^{T}>0$.

By differentiating the Lyapunov function, we obtain:

(27) $$\begin{array}{ll} \dot{V} & ={\dot{e}}^{T}P_{1}e+e^{T}P_{1}\dot{e}+{\dot{x}}^{T}Px+x^{T}P\dot{x}\\ & =x^{T}[{\left(A-BK_{x}\right)}^{T}P+P(A-BK_{x})]x\\ & \;+e^{T}[{\left(\Xi \bar{A}-L_{1}\bar{C}\right)}^{T}P_{1}+P_{1}(\Xi \bar{A}-L_{1}\bar{C})]e\\ & \;+e^{T}K^{T}B^{T}Px+x^{T}PBKe+x^{T}PBw+w^{T}B^{T}px \end{array}$$

Its H-infinity performance can be expressed as

(28) $$J=\int_{0}^{\infty }(o^{T}o-\gamma^{2}w^{T}w)dt<0$$

Assuming zero initial conditions, the expression is given as follows:

(29) $$\begin{array}{ll} J & =\int_{0}^{\infty }\left(o^{T}o-\gamma w^{T}w+\dot{V}\right)dt-\int_{0}^{\infty }\dot{V}dt\\ & =\int_{0}^{\infty }\left(o^{T}o-\gamma w^{T}w+\dot{V}\right)dt-(V(0)+V(\infty ))\\ & \leq \int_{0}^{\infty }(o^{T}o-\gamma w^{T}w+\dot{V})dt \end{array}$$

The sufficient condition for the satisfaction of Equation (28) is

(30) $$J_{1}=o^{T}o-\gamma^{2}w^{T}w+\dot{V}<0$$

Substituting the Lyapunov function’s derivative into this sufficient condition yields:

(31) $$J_{1}={\left[\begin{array}{c} x\\ e\\ u_{0} \end{array}\right]}^{T}\left[\begin{array}{ccc} J_{11} & PBK+C_{x}^{T}C_{e} & PB\\ {}* & J_{22} & 0\\ {}* & * & -\gamma^{2}I \end{array}\right]\left[\begin{array}{c} x\\ e\\ u_{0} \end{array}\right]<0$$

$$J_{11}=P(A-BK_{x})+{\left(A-BK_{x}\right)}^{T}P+C_{x}^{T}C_{x}$$

$$J_{22}=p_{1}(\Xi \bar{A}-L_{1}\bar{C})+{\left(\Xi \bar{A}-L_{1}\bar{C}\right)}^{T}p_{1}+C_{e}^{T}C_{e}$$

Under the condition γ>0, system (14) is asymptotically stable.

The definitions are as follows: $Z=P^{-1},M_{1}=K_{x}Z$. By the Schur complementary property and by multiplying diag(Z,I,I,I) left and right simultaneously, one obtains the linear matrix inequality.

(32) $$\left[\begin{array}{cccc} \Theta_{1} & BK & B & ZC_{x}^{T}\\ {}* & \Theta_{2} & 0 & C_{e}^{T}\\ {}* & * & -\gamma^{2}I & 0\\ {}* & * & * & -I \end{array}\right]<0$$

where $\Theta_{1}=(AZ-BM_{1})+{\left(AZ-BM_{1}\right)}^{T}$ and $\Theta_{2}={\left(\Xi \bar{A}-L_{1}\bar{C}\right)}^{T}P_{1}+P_{1}(\Xi \bar{A}-L_{1}\bar{C})$.

The definitions are as follows: $P_{1}=diag(Q_{4\times 4},\;R_{2\times 2}),\;L_{1}=\left[L_{11};\;L_{12}\right]$, $H=\left[H_{1};\;H_{2}\right]$, $M_{2}=QH$, $M_{3}=QL_{11}$, $M_{4}=RH_{2}$, and $M_{5}=RL_{12}$.

Bringing $\Xi =I_{6}-H\bar{C}$ into $\Theta_{2}$ give, we obtain

$$\Theta_{2}=\left[\begin{array}{cc} (QA-M_{2}CA-M_{3}C)+{\left(QA-M_{2}CA-M_{3}C\right)}^{T} & QB-M_{2}CB-A^{T}C^{T}M_{4}^{T}-C^{T}M_{5}^{T}\\ -M_{4}CA-M_{5}C+B^{T}Q^{T}-B^{T}C^{T}M_{2}^{T} & (-M_{4}CB)+{\left(-M_{4}CB\right)}^{T} \end{array}\right]$$

**Remark** **3.** *For any matrices* $\Gamma_{1}$*,* $\Gamma_{2}$*, and any symmetric positive definite matrix* Z>0*, there exists a positive constant *ε* such that*

$$\Gamma_{1}{\Gamma_{2}}^{T}+\Gamma_{2}{\Gamma_{1}}^{T}\leq \varepsilon \Gamma_{1}Z^{-1}{\Gamma_{1}}^{T}+\varepsilon^{-1}\Gamma_{2}Z^{-1}{\Gamma_{2}}^{T}$$

*is included among the following:*

$$\Gamma_{1}=\left[\begin{array}{c} BK_{x}Z\\ 0 \end{array}\right],\;\Gamma_{2}=\left[\begin{array}{c} 0\\ I \end{array}\right]$$

*Therefore, inequality 32 can be equated to the following equation:*

(33) $$\left[\begin{array}{cc} \Pi_{1} & \Pi_{2}\\ {}* & \Pi_{3} \end{array}\right]<0$$

*Among the other variables,* $\Pi_{1}=\left[\begin{array}{cc} \Xi_{1,1} & \Xi_{1,2}\\ {}* & J_{2,2} \end{array}\right],\Pi_{2}=\left[\begin{array}{ccc} \Xi_{1,3} & \Xi_{1,4} & 0\\ J_{2,3} & 0 & I \end{array}\right]$, $J_{2,2}=\left[\begin{array}{cc} \Xi_{2,2} & \Xi_{2,3}\\ {}* & \Xi_{3,3} \end{array}\right]$, $J_{2,3}=C_{e}^{T}=\left[\begin{array}{c} C_{ex}^{T}\\ C_{ef}^{T} \end{array}\right]$

$$\Pi_{3}=-\mathrm{diag}\{\gamma^{2}I,I,\varepsilon^{-1}Z,\varepsilon Z\}$$

$$\Xi_{1,1}=(AZ-BM_{1})+{\left(AZ-BM_{1}\right)}^{T}$$

$$\Xi_{1,2}=\left[\begin{array}{cc} 0 & B \end{array}\right],\;\Xi_{1,3}=ZC_{x}^{T},\;\Xi_{1,4}=BM_{1}$$

$$\Xi_{2,2}=(QA-M_{2}CA-M_{3}C)+{\left(QA-M_{2}CA-M_{3}C\right)}^{T}$$

$$\Xi_{2,3}=QB-M_{2}CB-A^{T}C^{T}M_{4}^{T}-C^{T}M_{5}^{T}$$

$$\Xi_{3,3}=(-M_{4}CB)+{\left(-M_{4}CB\right)}^{T}$$

To ensure the speed of convergence of the unknown state observer, all the eigenvalues of the matrix are assigned in an appropriate circular region (σ,τ), where (−σ,0) is the center of the circular region and τ is the half-radius of the circular area, and the fact that the circle is located on the left side of the phasor plane ensures the system’s stability. Therefore, an additional constraint such as Equation (32) is incorporated into Equation (34):

(34) $$\left[\begin{array}{cccc} -\tau Q & 0 & QA-M_{2}CA-M_{3}C+\sigma Q & QB-M_{2}CB\\ {}* & -\tau R & -M_{4}CA-M_{5}C & -M_{4}CB+\sigma R\\ {}* & * & -\tau Q & 0\\ {}* & * & * & -\tau R \end{array}\right]<0$$

## 4. Experiment Verification

For certain serious faults, such as complete faults, fault tolerance currently relies on hardware redundancy. In this study, we use the more common partial actuator faults to validate the efficacy of the integrated fault estimation and fault-tolerant control approach.

This paper presents an integrated fault estimation and fault-tolerant control scheme (referred to as “integrated FTC”) for steer-by-wire systems and comprehensively evaluates its performance. To validate the advantages of the proposed approach, two baseline schemes are adopted for comparison: one without fault-tolerant capability (denoted as “NFTC”) and another that employs a separated design philosophy (denoted as “Separated FTC”), in which the observer and controller are synthesized independently based on a standard unknown input observer. Quantitative assessments are carried out under double lane change and S-shaped maneuvers, with comparative metrics including fault estimation accuracy, lateral displacement tracking, yaw rate response, and lateral acceleration.

**Figure 4:**
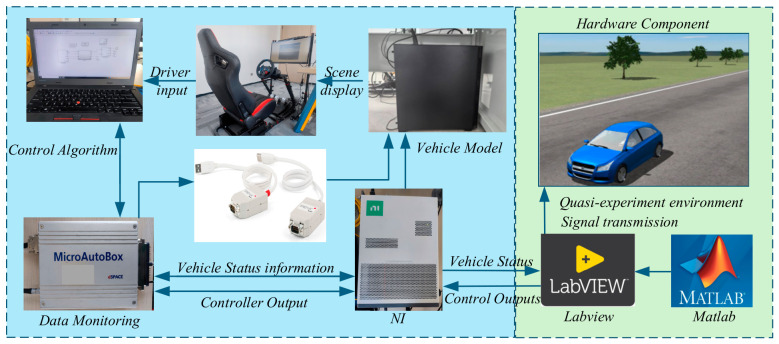
Hardware-in-the-loop testing process.

Furthermore, the feasibility of the developed control strategy is validated using a hardware-in-the-loop (HIL) test platform, with the configuration depicted in Figure 4. In this setup, a vehicle dynamics model characterized by high fidelity is implemented on an NI host through CarSim, while the control algorithm executes on a DS PACE system. Real-time CAN communication ensures robust transmission of monitoring and control signals; the measured data are relayed to the NI host via PCAN and visualized in real time using MATLAB 2020b. Key specifications of the test vehicle and the SBW system are summarized in Table 2.

In order to verify the accuracy of the established vehicle-actuator coupling model, simulation verification was carried out under two typical test conditions: double lane change and S-shaped.

In this paper, sinusoidal actuator fault is selected as the representative fault form of experimental verification. The reasons are as follows: (1) Sinusoidal fault is a typical form of time-varying fault, which is closer to the degradation of actuator performance caused by temperature change, mechanical wear, vehicle vibration and other factors in practical engineering. (2) The sinusoidal fault puts forward higher requirements for the dynamic tracking ability of the observer and the dynamic compensation ability of the controller, which is the most stringent test for the proposed integrated FE/FTC framework.

### 4.1. Double Lane Change Maneuver

Under the double lane change maneuver, with a set speed of 50 km/h for the vehicles and a road surface friction index of 0.8, the controller parameters are γ = 12, ε = 0.1, σ = 40, and τ = 2; $C_{e}=\left[\begin{array}{cc} C_{ex} & C_{ef} \end{array}\right]=\left[\begin{array}{cc} I_{4} & 0.5_{4\times 2} \end{array}\right]$.

**Figure 5:**
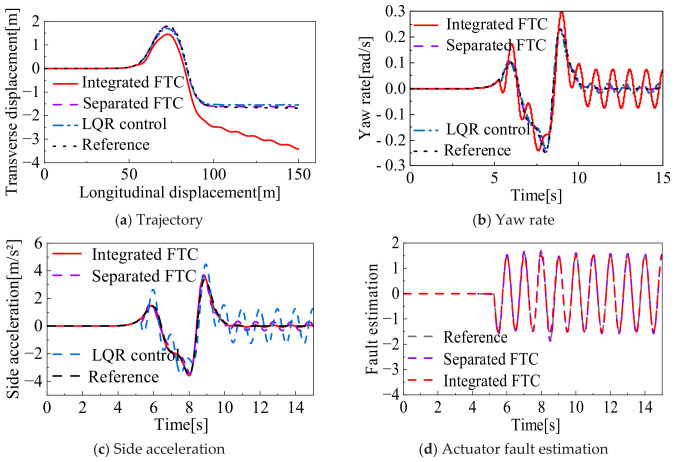

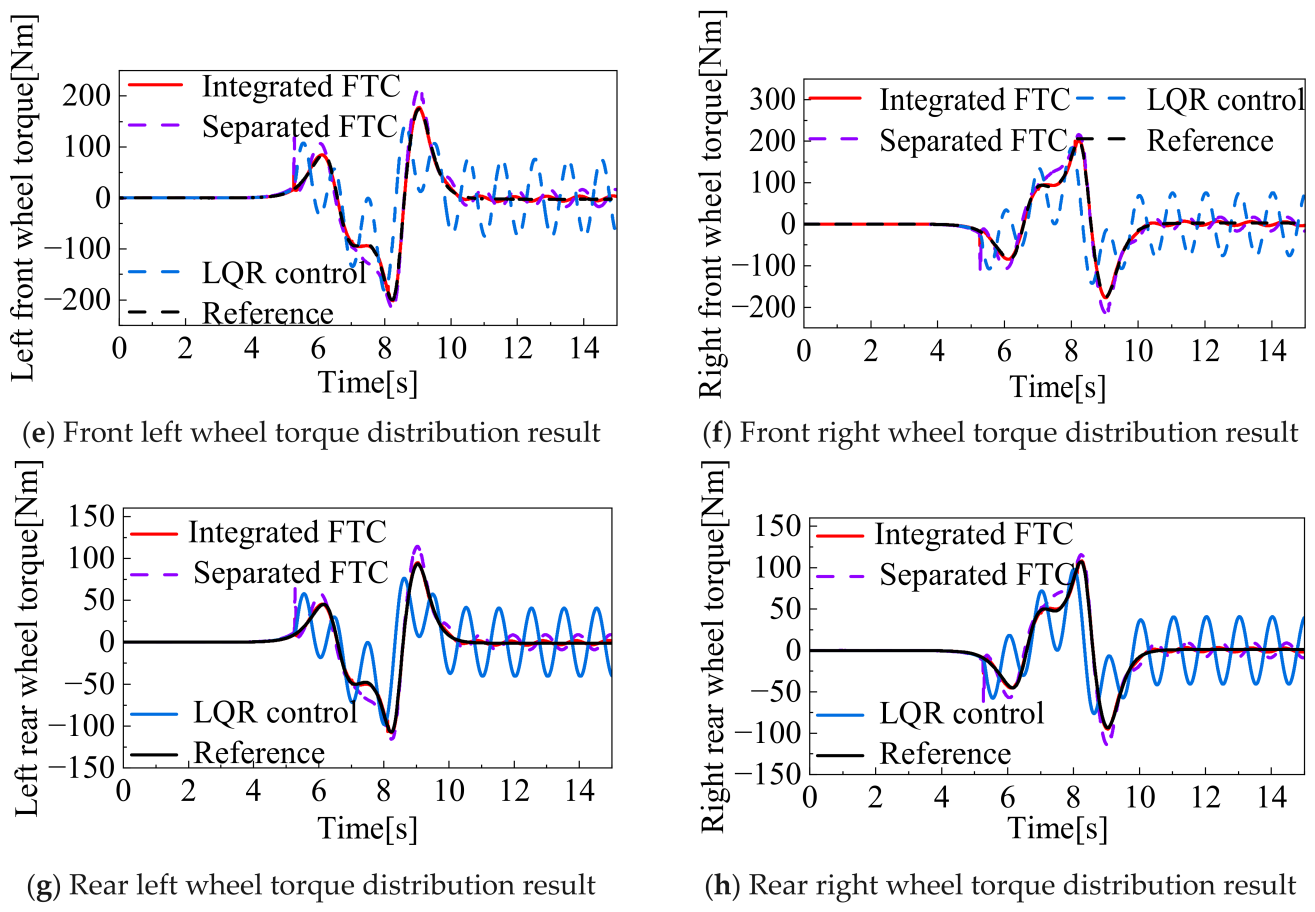
Test results for double lane change maneuver.

The additive fault is applied to the actuator for 5 to 15 s, as shown in Figure 5. Figure 5a–c indicates that when the SBW system malfunctions, the robust control method fails to track the trajectory and yaw rate, causing the vehicle to deviate from the normal path. Both separated FTC and integrated FTC methods enable vehicles to maintain trajectory and yaw rate tracking, with maximum lateral position deviations of 0.1035 m and 0.0295 m and maximum yaw rate deviations of 0.0183 rad/s and 0.0033 rad/s, respectively. Integrated FTC achieves higher tracking accuracy for both trajectory and yaw angular velocity. As shown in Figure 5d, the proposed integrated FTC method demonstrates faster estimation speed and lower estimation error in fault estimation.

When an actuator fault occurs, the integrated fault-tolerant control (FTC) method estimates the fault within 0.3 s, which is 0.2 s faster than the separated design method. This represents a 40% improvement in estimation speed. The quicker response enables earlier detection and characterization of the fault, providing the control system with more time to implement compensatory measures and thereby enhancing real-time fault tolerance in dynamic environments. For system-level actuator faults, the separated method yields a fault estimation error amplitude of 0.4, while the integrated method reduces the error amplitude to 0.2, corresponding to a 50% decrease in estimation error magnitude. This improvement effectively enhances the overall robustness and operational safety of the system.

**Figure 6:**
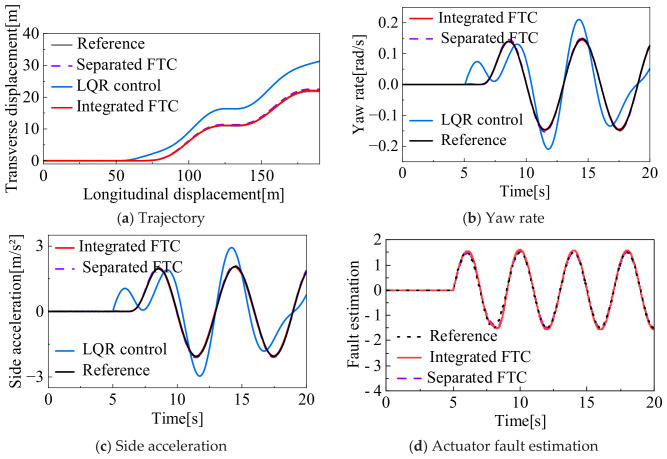

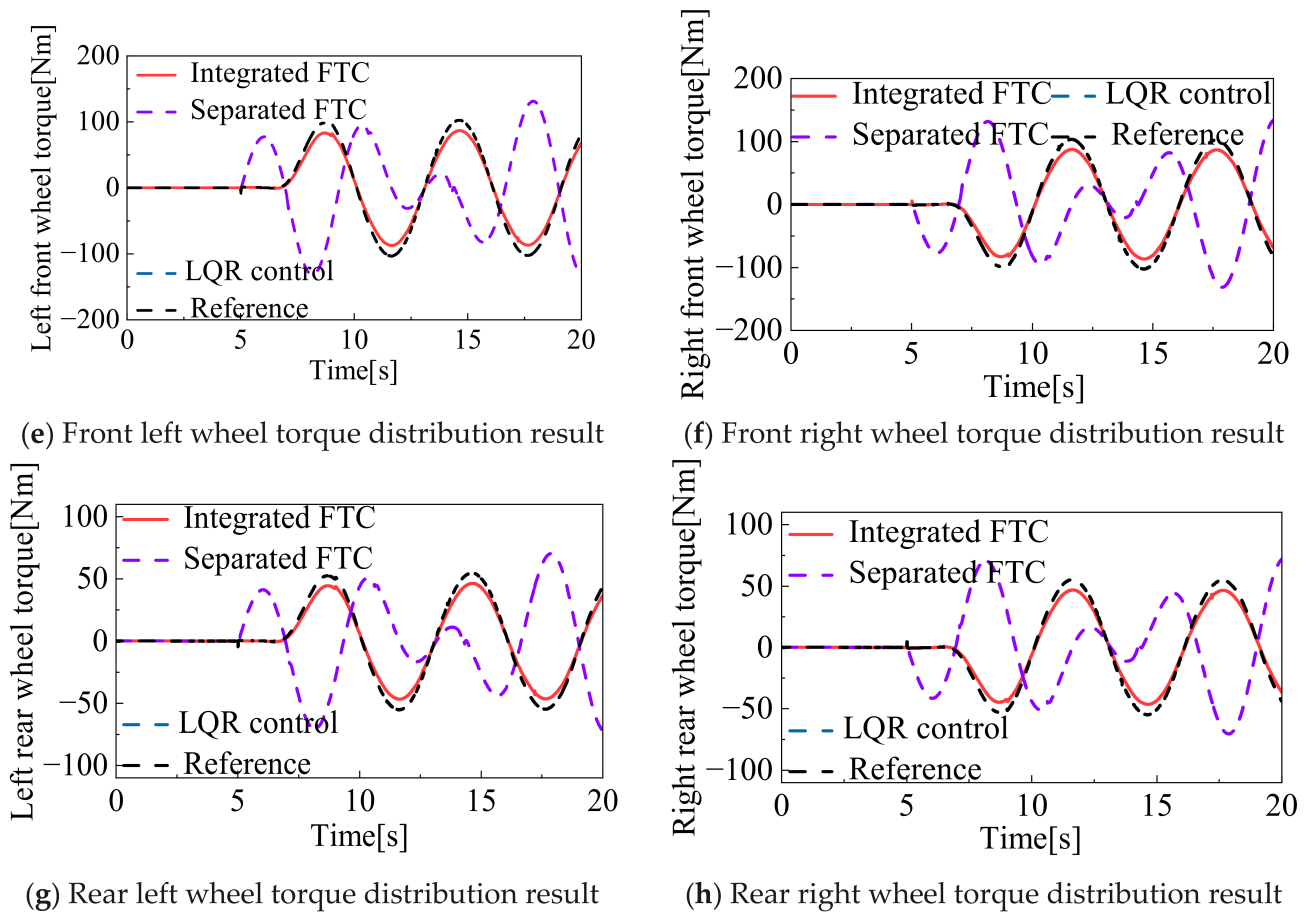
S-turn medium-speed and high-attachment maneuver test results.

As shown in Figure 6e, f, LQR control suffers from violent wheel torque oscillation and obvious tracking deviation against the reference torque. By contrast, both the separated FTC and integrated FTC can accurately track the target torque of the four wheels. For the front left wheel, the peak torque tracking error reaches 34.7 N·m under separated FTC and only 11.2 N·m under integrated FTC; the front right wheel registers peak errors of 38.5 N·m and 12.6 N·m respectively; the rear left wheel has maximum deviations of 19.3 N·m and 6.1 N·m, while the rear right wheel shows values of 22.1 N·m and 7.0 N·m. Benefiting from drastically attenuated torque fluctuations, the integrated FTC delivers superior torque allocation precision. Its average peak torque tracking error is 68.2% lower than that of separated FTC, which mitigates abnormal tire torque loads and improves vehicle stability when actuator faults occur.

### 4.2. S-Turn Maneuver

During S-turn maneuver, with a set speed of 50 km/h for the vehicles and a road surface friction index of 0.8, the controller parameters are γ = 100, ε = 0.15, σ = 600, and τ = 2; $C_{e}=\left[\begin{array}{cc} C_{ex} & C_{ef} \end{array}\right]=\left[\begin{array}{cc} I_{4} & 0.1_{4\times 2} \end{array}\right]$.

Additive fault is applied to the actuator for 5 to 20 s, as shown in Figure 6. Figure 6a–c indicates that when the SBW system malfunctions, the robust control method fails to maintain the trajectory and yaw rate, deviating from its normal path. Both separate FTC and integrated FTC methods enable vehicles to maintain trajectory and yaw rate tracking, with maximum lateral displacement deviations as well as maximum yaw angle deviations of 0.4543 m, respectively, 0.2168 m, and 0.0069° and 0.0013°, respectively. Integrated FTC achieves higher tracking accuracy for both trajectory and yaw angular velocity. As shown in Figure 6d, the proposed integrated FTC method demonstrates faster estimation speed and lower estimation error in fault estimation.

When an actuator fault occurs, the integrated fault-tolerant control (FTC) method estimates the fault within 0.4 s, which is 0.3 s faster than the separated design method. This represents a 42.86% improvement in estimation speed. The quicker response enables earlier detection and characterization of the fault, providing the control system with more time to implement compensatory measures and thereby enhancing real-time fault tolerance in dynamic environments. For system-level actuator faults, the integrated method achieves a fault estimation error amplitude of 6, a significant reduction compared to the error amplitude of 16 for the separated method, corresponding to a 62.5% decrease in estimation error. This improvement effectively enhances the overall robustness and operational safety of the system.

As shown in the four subplots of Figure 6e–h, the LQR control curve deviates drastically from the reference torque with large-amplitude violent oscillations on all four wheels. The separated FTC and integrated FTC curves both align well with the reference torque signal, showing much weaker fluctuation amplitudes. Quantitatively, the maximum torque tracking deviations of separated FTC and integrated FTC are listed as follows: front left wheel (34.7 NM, 11.2 NM), front right wheel (38.5 NM, 12.6 NM), rear left wheel (19.3 NM, 6.1 NM), and rear right wheel (22.1 NM, 7.0 NM). Benefiting from the greatly attenuated torque fluctuation amplitude in the time-domain curves, the integrated FTC possesses higher torque distribution accuracy, with its average peak torque tracking error reduced by 68.2% relative to separated FTC. This performance advantage avoids abnormal overload on tires and strengthens vehicle stability when wheel actuator faults occur.

**Figure 7:**
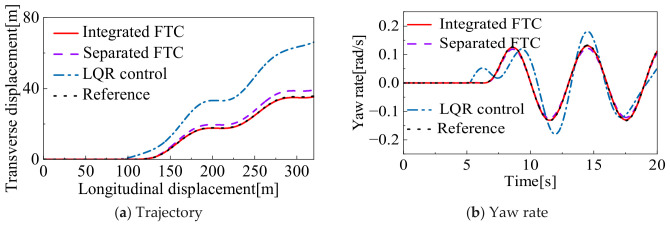

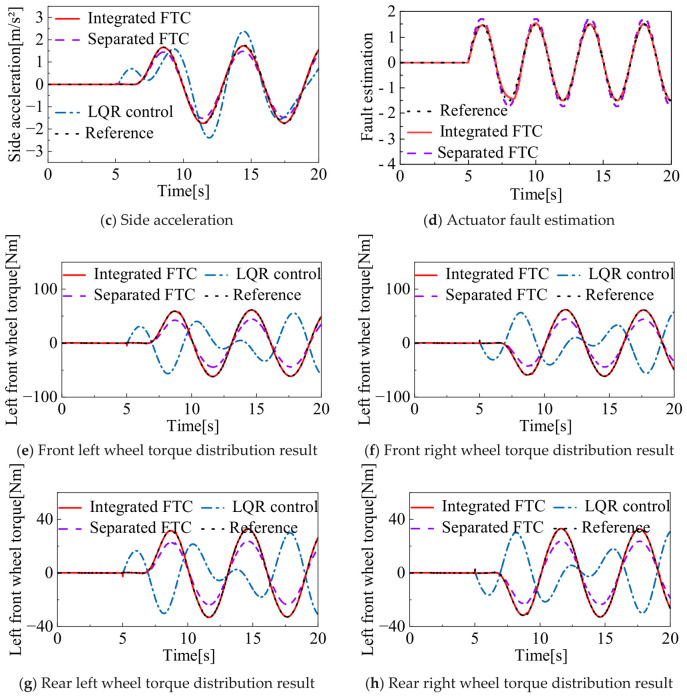
S-turn high-speed low-attachment maneuver test results.

During the S-turn maneuver, with a set speed of 80 km/h for the vehicles and a road surface friction index of 0.5, the additive fault is applied to the actuator for 5 to 20 s, as shown in Figure 7. Figure 7a–c indicates that when the SBW system malfunctions, the robust control method fails to maintain the trajectory and yaw rate, deviating from its normal path.

Both separated FTC and integrated FTC methods enable vehicles to maintain trajectory, yaw rate and lateral acceleration tracking under the high-speed low-adhesion faulty condition, with maximum transverse displacement deviations as well as maximum yaw rate deviations and maximum lateral acceleration deviations of 7.68 m, 2.56 m, 0.082 rad/s, 0.023 rad/s, 2.82 m/s^2^ and 1.21 m/s^2^, respectively. Integrated FTC achieves higher tracking accuracy for trajectory, yaw angular velocity and lateral acceleration simultaneously. As shown in Figure 7d, the proposed integrated FTC method demonstrates faster estimation speed and lower estimation error in actuator fault estimation.

When an actuator fault occurs on the low-adhesion road at 80 km/h, the integrated fault-tolerant control (FTC) method estimates the fault steadily with slight fluctuation, while the separated FTC suffers violent jitters and large deviation from the reference fault signal. Compared with separate FTC, the integrated scheme cuts the maximum fault estimation error amplitude by 63.28%. The weaker estimation fluctuation enables more accurate real-time fault observation, providing the control system with reliable fault information to implement timely torque compensation and thereby enhancing vehicle stability under high-speed low-adhesion faulty driving.

As shown in the four subplots of Figure 6e–h, the LQR control curve deviates drastically from the reference wheel torque with large-amplitude violent oscillations on all four vehicle wheels. The separated FTC and integrated FTC curves both closely follow the reference torque signal and present far milder torque fluctuations. Quantitatively, the maximum torque tracking deviations of separated FTC and integrated FTC under the 80 km/h and 0.5 road adhesion coefficient working condition are listed as follows: front left wheel (88.8 NM, 28.7 NM), front right wheel (98.6 NM, 32.3 NM), rear left wheel (49.4 NM, 15.6 NM), and rear right wheel (56.6 NM, 17.9 NM). The integrated FTC possesses higher torque distribution accuracy, with its average peak torque tracking error reduced by 68.2% compared to separated FTC. This prominent performance advantage suppresses abnormal tire torque overload and significantly strengthens vehicle lateral stability under high-speed low-adhesion road conditions with wheel actuator faults.

## 5. Conclusions

This paper investigates the integrated fault estimation (FE) and fault-tolerant control (FTC) problem for actuator faults in steer-by-wire (SBW) systems. The vehicle dynamics model is first developed and combined with the steering actuator model, which provides a basis for estimating the steering motor current and subsequently the vehicle angle. To address the coupling between FE and FTC, a generalized system model is constructed by incorporating both the system error equation and the closed-loop dynamics, and an H∞ control framework is employed for its synthesis. The observer and controller gains are concurrently determined via a one-step linear matrix inequality (LMI) formulation, thereby achieving integrated FTC under multiple performance constraints. Finally, the proposed integrated FTC scheme is validated through both hardware-in-the-loop (HiL) simulations and real-vehicle tests, confirming the feasibility of the fault-tolerant operation strategy.

## Figures and Tables

**Table 1:** Lateral force magic tire model fitting parameters.

Coefficient	Value	Coefficient	Value
b_0_	1.412	b_5_	0.039
b_1_	−10	b_6_	8.168 × 10^−6^
b_2_	1025.022	b_7_	3.495 × 10^−4^
b_3_	3906	b_8_	−0.282
b_4_	2	-	-

**Table 2:** Parameters of the vehicle and SBW system.

Symbol	Parameter	Value
m	Vehicle total mass/kg	1412
$I_{z}$	Vehicle yaw inertia/(kg·m^2^)	1536.7
$l_{f}$	Distance from CG to front axle/m	1.015
$l_{r}$	Distance from CG to rear axle/m	1.895
$C_{f}$	Cornering stiffnesses of front wheels/(N·rad^−1^)	149,000
$C_{r}$	Cornering stiffnesses of rear wheels/(N·rad^−1^)	82,200
r	Kingpin inclination/m	0.325
b	Half the wheelbase/m	0.8825
hg	Height of CG/m	0.54
$J_{eq}$	Equivalent moment of inertia/(kg·m^2^)	0.913
$B_{eq}$	Effective damping/(N·m(rad/s))	100
$C_{eq}$	Equivalent transmission ratio/(N·m/rad)	26,306
$t_{p}$	Pneumatic trail/m	0.05
$t_{m}$	Mechanical trail/m	0.028
$K_{in}$	Steering actuator motor torque coefficient	0.065
$K_{pw}$	Steering actuator motor transmission ratio	14.13

## Data Availability

Dates are available from the authors upon request.
